# Unusual Location of Bone Tumor Easily Misdiagnosed: Distal Radius Osteosarcoma Treated as Osteomyelitis

**DOI:** 10.7759/cureus.19905

**Published:** 2021-11-25

**Authors:** Muath Mamdouh Mahmod Al-Chalabi, Izzeddin Jamil, Wan Azman Wan Sulaiman

**Affiliations:** 1 Reconstructive Sciences Unit, Universiti Sains Malaysia (USM), Kota Bharu, MYS

**Keywords:** osteomyelitis, wrist osteosarcoma, rare osteosarcoma, distal radius osteosarcoma, osteosarcoma

## Abstract

Despite being the most common primary malignant bone tumor in children and adolescents, the presence of osteosarcoma at the wrist is infrequent; only less than 1% of osteosarcomas arise in the distal radius. The clinical presentation may mimic common musculoskeletal problems or benign lesions such as osteomyelitis, and a high index of suspicion is necessary so that the treating surgeon does not miss such lesions. We reported a case treated initially as osteomyelitis before being diagnosed as distal radius osteosarcoma. We conclude that an unusual location of osteosarcoma may be easily misdiagnosed, and therefore, osteosarcoma should be considered one of the main differential diagnosis in such cases until proven otherwise.

## Introduction

Osteosarcoma, Ewing sarcoma, and chondrosarcoma are all primary bone malignancies. They account for less than 1% of all malignancies diagnosed annually, yet they are associated with high morbidity and death. Osteosarcoma is the most prevalent type of bone cancer, accounts for roughly two-thirds of all occurrences [1,2]. Osteosarcoma is predominantly a childhood illness, with a slight increase in incidence among older people. It is the third most common childhood malignancy, with a median incidence at 12 years of age for girls and 16 years for boys [1]. Malignant primitive mesenchymal cells develop into osteoblasts, which generate a malignant osteoid matrix, which causes osteosarcoma. Osteosarcomas can grow in any bone, but they are most commonly found in the metaphysis of long bones. The proximal humerus, distal femur, and proximal tibia account for about 60% of all cases [1].

The most common symptoms of bone malignancies are localized or regional pain with overlaying tenderness and restricted range of motion. These signs and symptoms could be mistaken for common musculoskeletal problems [1]. Osteomyelitis is a common benign illness that mimics the symptoms of osteosarcoma. On the other hand, osteosarcoma of the wrist is extremely rare; only around 1% of osteosarcomas occur in the distal radius, according to studies [3]. A correct diagnosis is essential to achieve proper and definitive treatment. The most critical information physicians use in generating a pertinent differential diagnosis for bone tumors is clinical evaluation and radiologic studies. Histologic examination of the tumor and relevant additional laboratory studies are then used to conclude the final diagnosis [4]. The time of diagnosis is crucial because it determines treatment choice and the feasibility of limb salvage, which has permanent physical and psychological implications.

## Case presentation

**Figure 1:**
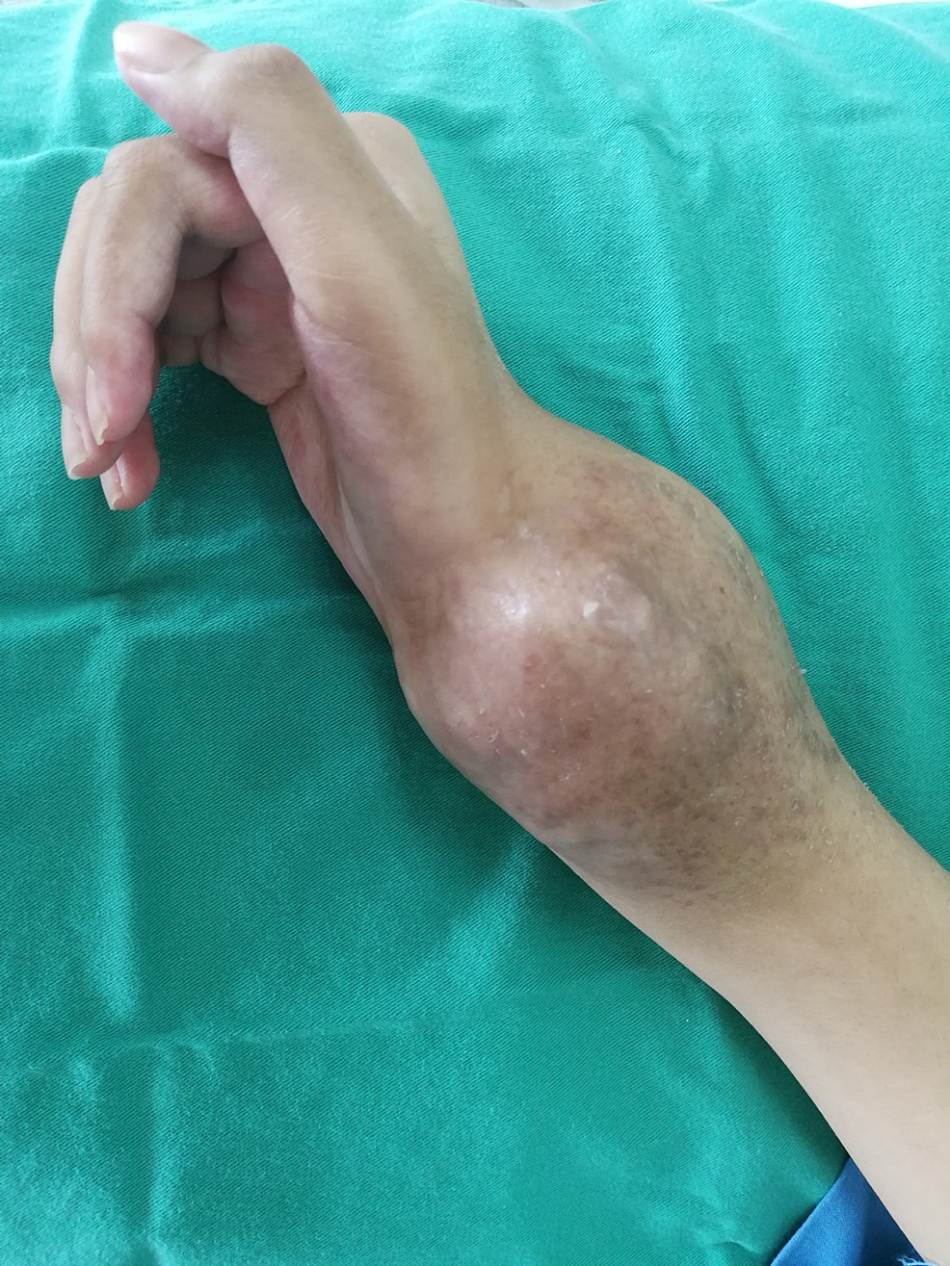
Diffuse circumferential swelling over the right wrist joint.

A 15-year-old Malay girl was referred to our center with a right lunate bone dislocation after being treated as osteomyelitis of the right distal ulna and radius for six months. Initially, she presented with right wrist pain, which worsened after lifting heavy objects, with increasing pain intensity, especially at night. However, the swelling started at the wrist and gradually increased in size, especially in the last three months. She denied any history of recent trauma or falls. Her condition was associated with a significant loss of weight over the previous six weeks prior to admission. Physical examination showed diffuse, circumferential tender swelling at the right wrist joint. Wrist motion was markedly restricted (Figure 1).

**Figure 2:**
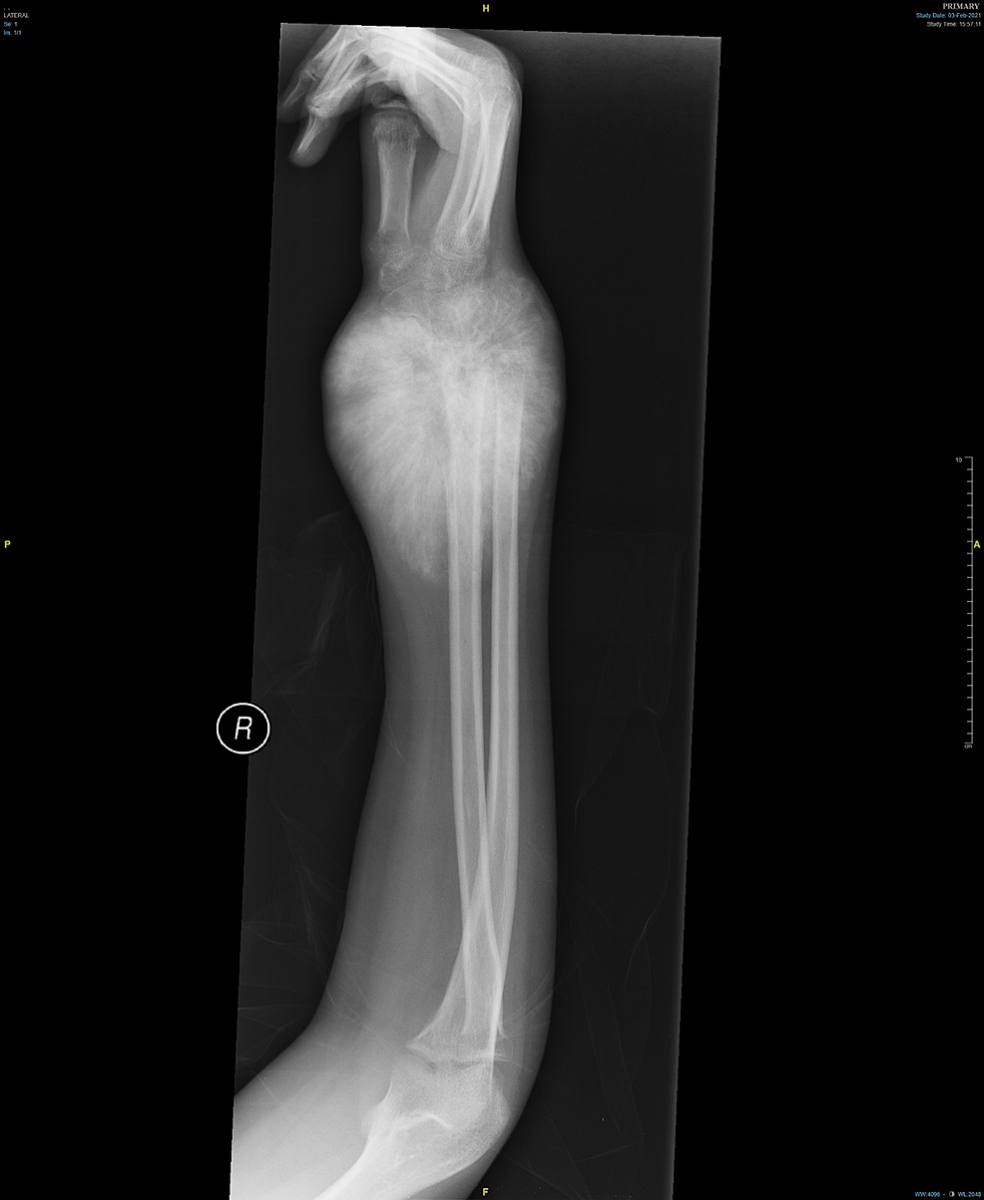
X-ray of the right upper limb shows moth-eaten appearance of distal radius metaphyseal region, lytic lesion, and cortex breakage at the radius styloid.

Radiograph of the right wrist showed a moth-eaten appearance of the distal radius at the metaphyseal region, lytic lesion, and cortex breakage at the radius styloid, a narrow zone of transition, increased soft tissue shadow, and thinning of the cortex (Figure 2).

**Figure 3:**
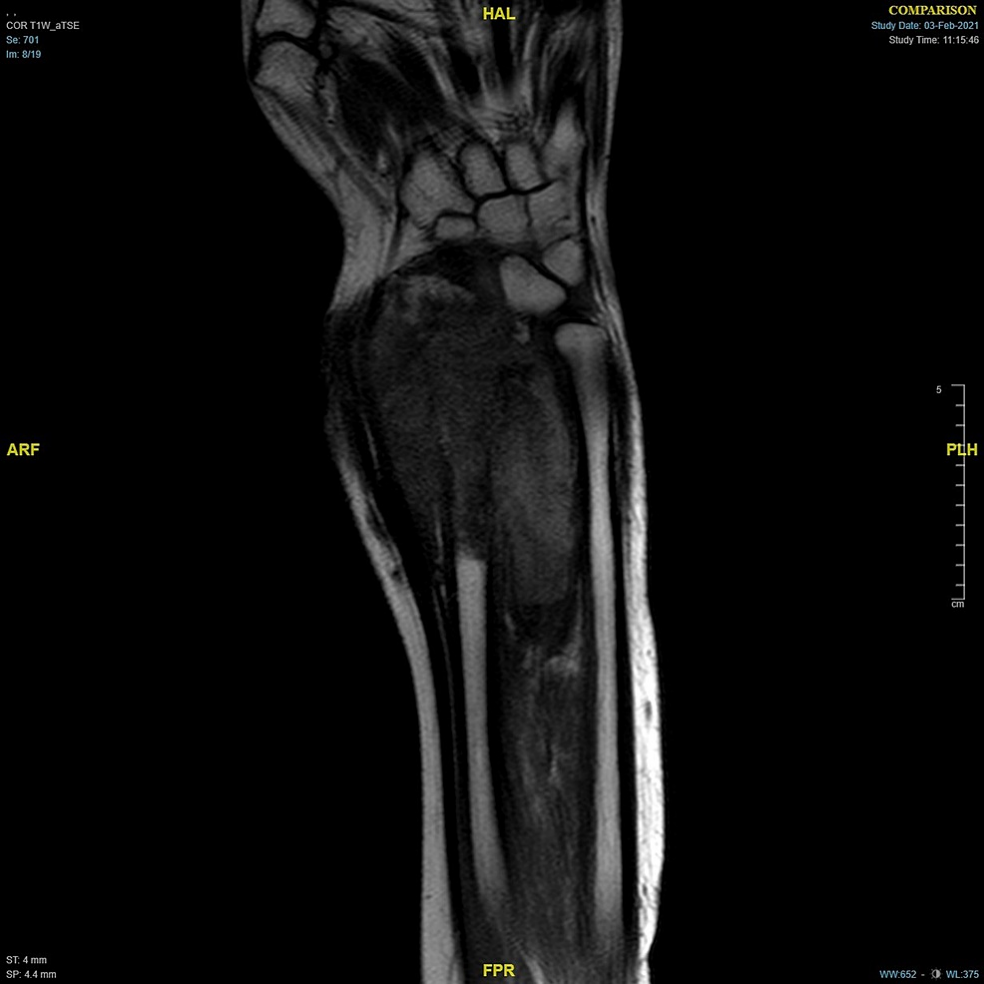
MRI of the right upper limb shows soft tissue mass occupying the distal right radius and ulna with extension to the carpal bones.

Magnetic resonance imaging (MRI) showed a soft tissue mass occupying the distal right radius and ulna, extending the carpal bones. This mass measures approximately 7.5 cm x 7.4 cm x 8.8 cm (Figure 3), suggesting a high-grade malignant tumor grown widely into the soft tissue from intramedullary bone. Lung metastasis appeared on chest computed tomography. A bone true-cut and core needle biopsy histopathology were consistent with osteosarcoma. She received neoadjuvant chemotherapy, but she did not show any meaningful improvement. However, she underwent limb salvage and wide resection of the right distal ulna, radius, and first row of carpal bones. Tendons and nerve repair were performed after reconstruction of the right upper limb with a free osteocutaneous fibula flap.

## Discussion

With an incidence of 0.3-0.5 per 100,000, osteosarcoma is the most frequent non-hematopoietic primary malignant bone tumor [5]. According to studies, the distal radius is a common skeletal site for primary bone tumors, but not for osteosarcomas, as only 1% of osteosarcomas are found in the distal radius [6,7]. Uribe-Botero et al. [8] investigated 243 cases with primary osteosarcoma of bone, and they found (49.8%) of tumors arising in the femur to be the first most frequent site, then tibia to be the second most common site, with 47 cases (19.3%), while only one case of distal radius osteosarcoma is recorded. Primary bone tumors are uncommon neoplasms with a wide range of clinical signs [4]. The most common signs of bone cancer are regional or localized pain with overlaying tenderness and restricted range of motion. These signs and symptoms could be mistaken for common musculoskeletal injuries. Bony malignancies can cause nighttime pain, pathological fractures, and joint dislocation; however, these symptoms are not common. Because of the aggressive nature of bone cancers, an accurate diagnosis is crucial to achieving effective and final treatment.

Radiography should be requested whenever there is persistent or recurring bone pain or soft tissue edema without evidence of trauma, as diagnosis delays are still common. Widhe [9] reported that if a radiograph was made at the first visit, the doctor’s delay averaged eight weeks compared with nineteen weeks when a radiograph was not made; therefore, clinical assessment and radiologic investigations are essential when making a differential diagnosis for bone cancers [10]. Histologic evaluation of the tumor and necessary additional laboratory procedures are then performed to determine the final diagnosis. Though, professionals should have a working diagnosis or a list of relevant differential diagnosis before submitting the patient to any invasive procedure.

Generally, female patients delay for a lengthy period before consulting a doctor, and being less muscular made it easier for the doctor to notice a palpable mass. These factors may describe the apparent difference between males and females about the delay in diagnosis [9]. The delay between the patient's first noted symptoms and the first medical visit may be impossible to change, but shortening the period from the first medical check-up to figure out an accurate diagnosis is the only way to reduce the total delay. Widhe [9] mentioned that three factors commonly result in delayed diagnosis of osteosarcoma; one of them is that the management for another diagnosis is ongoing for too long, although the variation of the clinical picture from what could be anticipated from the original diagnosis. This factor is critical because it may result in a worse prognosis and extensive loss.

## Conclusions

Osteosarcoma of the distal radius is a rare tumor and clinically may mimic other common musculoskeletal illnesses. Considering osteosarcoma in an early stage of any boney swelling in an unusual location reduces the chance of misdiagnosis. It allows limb salvage with complete clearance of the lesion and a margin of normal tissue, preserving the essential structures and joint surfaces to maintain wrist stability and function.
